# Editorial: Leadership in teamwork: enhancing rehabilitation medicine best practice

**DOI:** 10.3389/fresc.2023.1270116

**Published:** 2023-10-18

**Authors:** Zaliha Binti Omar, Hitoshi Kagaya, Christine Mac Donell

**Affiliations:** ^1^ Department of Rehabilitation Medicine, University of Malaya, Kuala Lumpur, Malaysia; ^2^ Department of Rehabilitation Medicine, Fujita Health University, Toyoake, Japan; ^3^ Department of Rehabilitation Medicine, National Center for Geriatrics and Gerontology, Aichi, Japan; ^4^ Medical Rehabilitation and International Aging Services, CARF, Tuscon, AZ, United States

**Keywords:** leadership, teamwork, rehabilitation, person-centered care, systems thinking approach

**Editorial on the Research Topic** Leadership in teamwork: enhancing rehabilitation medicine best practice

The primary aim of this research topic was to bring together a collection of papers that focused on leadership and teamwork in Rehabilitation Medicine to shed light on approaches and key factors affecting outcomes of person-centered care and the impact on rehabilitative care in general. Our main goals were (1) Identification of effective leadership models in the teamwork of person-centered care that could bring forth innovative practices in rehabilitation, (2) To advocate for real world interest in providing studies and documents to develop a critical mass of data to enhance person-centered care, and, (3) To promote evidence-based information and education on effective leadership in person-centered teamwork rehabilitation practice.

We welcomed the submission of manuscripts that included, but not limited to, the following topics:

• Historical perspectives of leadership in rehabilitation practice.
• Models of leadership in rehabilitation teamwork and their attributes.
• Leadership experience in person-centered care rehabilitation practice.
• Person-centered care as a basis for modelling leadership roles in healthcare and or rehabilitation practice.
• Theoretical basis and thesis generated by the application of the various healthcare, medical, social, anthropological and other models specific to rehabilitation practice.
• Theoretical models of leadership in Rehabilitation Medicine.
• Theoretical models of teamwork in rehabilitation practice.

We attracted submissions on a diverse range of relevant subjects from three continents; two from Asia, two from Europe and one from North America. Taslim Uddin from Bangladesh submitted a short communication that highlights the challenges for team leadership in developing countries. These include curriculum course content, monitoring systems, infrastructure development, funding, attitude, education level of people with disabilities, ethics and disasters. From China, Ping Zhou brought out an emerging rehabilitation team member that we cannot do without currently, i.e., a rehabilitation engineer. From Germany, the submission from Thorsten Meyer et al., titled “Cooperative leadership as a condition for patient reported rehabilitation success”, presents qualitative evidence for the importance of interdisciplinary teamwork and collaborative leadership. From France, the pilot study from R. Lenoir dit Caron et al. shows that team-based exercise programs proved superior to personalized ones. Finally, from Canada, Gillian King et al. shared their experience innovating a transition strategy at a pediatric rehabilitation hospital. The key elements identified in team members serve as useful lessons on team dynamics.

These submissions are so diverse in their content, ranging from conventional and emerging team memberships, cohesiveness, and shared responsibilities to a person-centered exercise program. The relative scarcity of literature and submissions on the core principles of rehabilitation practice that this research topic called for implies rehabilitation has only just begun to nurture the current and future rehabilitation professionals in the field. Person-centered care, teamwork and leadership in rehabilitation are dynamically intertwined. Each of these unique components require consistent education of rehabilitation health care providers, data driven action and evidence-based practice. Rehabilitation, as conventionally practiced, is at a critical juncture requiring new skills, knowledge, data, and evidence to meet the exciting challenge of progression in the specialty.

Person-centered care can only be realized by consciously recognizing the person as the expert in their lives and incorporating that critical component in every step of the way throughout all aspects of rehabilitation. Persevering in person-centered care calls for a hard, soft, and critical systems thinking approach (1) that is designed and practiced to the point of it being second nature in rehabilitation practice. We need to deepen our actions through the hard systems in established frameworks including the United Nations' 2030 Agenda for Sustainable Development (2), the WHO Rehabilitation 2030: A Call for Action (3), the Cochrane definition of rehabilitation for research purposes (4), rehabilitation classifications that continue to grow, especially the International Classification of Functioning, Disability, and Health (5), and realization of the human functioning concept (Bickenbach et al.). Soft systems thinking approach can take care of multistakeholder real-world needs that are so often disregarded. Complex societal situations demand critical systems thinking approach to solve the most trivial daily issues encountered in rehabilitation practice.

Inter, multi and transdisciplinary teamwork is key to a cohesive effort in processing person-centered rehabilitative care. The expanse of knowledge and skills in the spectrum of evidence-based practice for health, wellness and functionality of individuals requires a team effort; woe to the practitioner who practices with a solo mindedness.

The art of practicing leadership in daily rehabilitation practice requires excellent communication skills, strategic planning, awareness of social determinants of health of persons receiving rehabilitation services plus cultural mores, and the inevitable environmental changes. A leader who is selfless opens doors and grants opportunities for rehabilitation team members to grow individually as well as a robust team. Effective and efficient person-centered care and teamwork that are perpetually practiced can propagate excellent leadership.

Turning data into action may seem a while away, but person-centered care and teamwork in rehabilitation are here to stay. Our creative innovative ways will be testimony to our adherence to these core principles in our specialty. Along with good leadership, they will remain important determinants of Rehabilitation Medicine best practice that will benefit the one in three people in the world who needs rehabilitation in the course of their disease or injury (6).
